# Hand hygiene multimodal strategy and the decrease on central line-associated bloodstream infection in a Brazilian neonatal intensive care unit

**DOI:** 10.1186/2047-2994-4-S1-P155

**Published:** 2015-06-16

**Authors:** R Richtmann, G Pereira, T Rodrigues, ACO Filhiolino, KM Gama, C Dealmeida Silva, L Dias

**Affiliations:** 1 Maternity Hospital Pro MatrePaulista, São Paulo, Brazil

## Introduction

Hand hygiene (HH) is the most important strategy to reduce healthcare associated infection. Central line associated bloodstream infection (CLABSI) is the most frequent and severe infection in the Neonatal intensive care unit (NICU).

## Objectives

In this work we describe the impact of World Health Organization (WHO) multimodal strategy implementation on the CLABSI incidence in the NICU.

## Methods

The WHO multimodal strategy was recommended by Sao Paulo State government in 2011 to all hospitals as a part of the campaign “Save lives: clean your “hands”. Our HH multi-professional team implemented several strategies as checking the infrastructure for HH, education and training, videos, HH playful campaigns, games, creation of a HH mascot. All actions were followed by continuous feed backs of compliance to HH and infection rates to healthcare workers and were coordinated by the infection control committee and supported by the hospital leadership.

A prospective study during 2011 to 2014 in a maternity hospital with 63 NICU beds in Sao Paulo city/Brazil.

## Results

The alcohol based product consumption for HH increased during this period from 33.8 ml to 90.0 ml/patient-day and , and the HH compliance increased from 63.0% to 84.0%, on the opposite the CLABSI incidence in the NICU reduced from 6.2 CLABSI/1000 Central Line (CL)-day to 1.5 CLABSI/1000 CL-day.

## Conclusion

The WHO multimodal strategy implementation wascrucialto guide the actions and systematization of the HH multiprofessional team. The commitment of a multiprofessional HH team was determinant to our outcome. We do have “saved lives” with all these actions and on 2014 both hospitals won the WHO Latin America HH excellence award as recognition of all our accomplishments.

## Disclosure of interest

None declared.

